# Concordance of an in-house 2-steps PCR-SSP and nanopore sequencing for HLA-B*57:01 and HLA-B*58:01 typing: a comparative study

**DOI:** 10.3389/fgene.2025.1649990

**Published:** 2025-09-25

**Authors:** Nattapong Intharuangrung, Chonticha Sirikul, Pattaraporn Nimsamer, Thidathip Wongsurawat, Auttachai Saejia, Nampeung Anukul

**Affiliations:** ^1^ Division of Transfusion Science, Department of Medical Technology, Faculty of Associated Medical Sciences, Chiang Mai University, Chiang Mai, Thailand; ^2^ Oxford Nanopore Centre of Excellence, Division of Medical Bioinformatics, Research Department, Faculty of Medicine Siriraj Hospital, Mahidol University, Bangkok, Thailand; ^3^ Siriraj Long-read Lab (Si-LoL), Faculty of Medicine Siriraj Hospital, Mahidol University, Bangkok, Thailand

**Keywords:** HLA, pharmacogenomics, nanopore, health, precision medicine, adverse drug reaction

## Abstract

This study reports an optimized in-house 2-step PCR-SSP assay for rapid, cost-effective detection of HLA-B*57:01 and HLA-B*58:01 in routine pharmacogenomics laboratory. This assay employs allele-specific primers positioned within exon 2–3 boundaries, validated *in silico* against common *HLA-B* alleles. Using 30 clinical DNA samples, our PCR workflow (<1 h) showed 100% concordance at 2-field resolution with Oxford Nanopore sequencing performed using ligation-based sequencing kit with PCR barcoding. Cohen’s kappa was 1.00 with 95% CI. The turnaround time and reagent cost per sample were reduced to 1 h of hands-on PCR time and USD 7 per sample, respectively. These do not include DNA extraction or gel electrophoresis analysis. This 2-step PCR-SSP offers a robust alternative for pharmacogenomic screening in resource-limited settings for detecting the HLA-B*57:01 and HLA-B*58:01.

## 1 Introduction

Abacavir is a drug used in the treatment of human immunodeficiency virus 1 (HIV-1) infection functioning as a nucleoside reverse transcriptase inhibitor (NRTI). It inhibits the activity of the virus reverse transcriptase enzyme and often used in combination with other antiviral drugs to enhance the antiviral effect (Pratt et al., 2012). Approximately 5% of patients prescribed abacavir may have adverse drug reactions or hypersensitivity responses within the first 6 weeks (Hetherington et al., 2001). Additionally, HIV-infected patients treated with abacavir who have the HLA-B*57:01 gene have a significantly higher risk of experiencing adverse drug reactions. These reactions often manifest as skin rashes, high fever, and difficulty breathing leading to severe cutaneous adverse reactions (SCAR) including Stevens-Johnson syndrome (SJS) and toxic epidermal necrolysis (TEN) which increase the risk of mortality (Mounzer et al., 2019; Ma et al., 2010). Allopurinol is a uric acid-lowering medication commonly used to treat gout and hyperuricemia due to its affordability and high effectiveness (Sukasem, 2009). This drug functions by inhibiting xanthine oxidase enzyme, thereby reducing uric acid production. Allopurinol can induce SCAR associated to the HLA-B*58:01 gene (Hung et al., 2005; Tassaneeyakul et al., 2009).

HLA-B*57:01 and HLA-B*58:01 alleles are part of the major histocompatibility complexes (MHC) class I group, found on the surface of white blood cells and tissues (Romphruk, 2004). These genes are globally distributed and show variation among variability among different ethnic groups. For example, the HLA-B*57:01 allele has been found 4%–10% of European descent, 2%–6% of North African descent, and 1%–7% of Southeast Asian descent (Gonzalez-Galarza et al., 2020). In Thai population, it can be found in only 1%–3% (Gonzalez-Galarza et al., 2020). This indicates that Thais have a chance of developing an abacavir-hypersensitivity reaction (ABC-HSR). HLA-B*58:01 allele has been found in up to 7.3% of Han Chinese, 5.5% of Southeast Asians, and is relatively rare in Japanese and European populations, found in only 0.6% and 0.8%, respectively (Tassaneeyakul et al., 2009). Therefore, to prevent adverse reactions after drug administration, it is advisable to screen for these alleles before starting treatment.

Screening for the HLA-B*57:01 and HLA-B*58:01 alleles, molecular biology approaches must be employed. Commonly used methods in pharmacogenomics laboratories include polymerase chain reaction with sequence-specific primer (PCR-SSP), polymerase chain reaction-sequence-specific oligonucleotide probes (PCR-SSOP), real-time polymerase chain reaction (Real-time-PCR), and Luminex™ technology. However, some techniques have limitations in terms of high costs. Therefore, developing in-house methods is still challenged because it can reduce the assay cost and still provide high-resolution results. It would be beneficial for laboratories offering pharmacogenetic testing services with limited resources and budget. Despite the availability of advanced techniques, a significant number of laboratories continue to rely on conventional PCR-SSP or PCR-SSP with high-resolution melting (HRM), primarily due to its economic advantages. Typically, it takes 1.5–3 h in PCR processing time. In an effort, an in-house 2-steps PCR-SSP has been developed in our facility, targeting a completion time of roughly 1 hour for detecting HLA-B*57:01 and HLA-B*58:01 alleles. To validate the performance of in-house 2-steps PCR-SSP, this study aimed to compare the testing results between in-house 2-steps PCR-SSP and the standard sequencing method specifically choosing nanopore sequencing for its future promising application in pharmacogenomics service.

## 2 Materials and methods

### 2.1 Study design

This study was a prospective observational study. The study aimed to validate the performance of an in-house 2-steps PCR-SSP against the sequencing method for the detection of HLA-B*57:01 and HLA-B*58:01 alleles.

### 2.2 DNA sample selection

DNA samples were selected from residual specimens used in routine HLA testing at Molecular Biology Laboratory, Division of Transfusion Science, Department of Medical Technology, Faculty of Associated Medical Sciences, Chiang Mai University. Purposive sampling was employed to select DNA samples with known presence of HLA-B*57:01 and/or HLA-B*58:01 alleles to ensure the inclusion of positive cases for concordance analysis. The sample size calculation was based on an expected prevalence p = 50% to reflect the intention of sample selection rather than estimate prevalence using the actual allele frequencies in the population. For reference, allele frequencies in Thai population reported in Allele Frequency Net database (http://www.allelefrequencies.net/hla.asp) are approximately 1.8% for HLA-B*57:01% and 7.7% for HLA-B*58:01 (Gonzalez-Galarza et al., 2020). These frequency values are provided for further random sampling. The sample size formula applied was; *n* = *Z* ^2^ × p × (1−p)/*d* ^2^, where Z is 1.96 for 95% confidence, p is the expected prevalence (here set to 0.5) and d is precision (0.05) (Pourhoseingholi et al., 2013). The calculated sample size based on this formula was approximately 385 samples. However, in this preliminary study, a smaller sample size was chosen due to the limited availability of positive samples with a total of 30 samples purposively selected to enable direct concordance analysis.

### 2.3 DNA extraction and preparation

DNA was extracted using QIAamp^®^ Blood Mini Kit (QIAGEN, Germany). DNA purity and concentration was then measured using the EON™microplate spectrophotometer (RI technologies ltd., Singapore) based on the principle of light absorption.

### 2.4 Primer design and modification

The original primer designs, as published by Martin et al. (2005) and Virakul et al. (2010) for HLA-B*57:01 and HLA-B*58:01, respectively, were modified to facilitate a 2-step PCR-SSP approach binding across exon 2–3 boundaries (patent pending). The reference sequences for HLA-B*57:01:*01:01* (IMGT/HLA Acc No: HLA00381) and HLA-B*58:01:*01:01* (IMGT/HLA Acc No: HLA00386) was retrieved from IMGT/HLA database. To guarantee effective and targeted amplification within a two-step cycling, the modifications focused on enhancing primer annealing and extension properties. All primers have T_m_ of 73 °C–75 °C and GC content 60%–68%. The *in silico* validation of primers was performed using multiple bioinformatics tools to confirm their specificity and efficiency. For specificity, the primers designed to target HLA-B*57:01 and HLA-B*58:01 alleles were validated using BLAST against the nucleotide BLAST in NCBI database with the Human RefSeq Gene database and megablast selection to confirm allele specificity and exclude cross-reactivity with non-target alleles or related sequences. For cross-reactivity within the HLA region, Probe and Primer Search Tool in IPD-IMGT/HLA database was used. Moreover, the primers were also assessed for melting temperature (Tm), GC content, self-complementarity, and the absence of potential primer-dimer formations using Primer3 software. These metrics ensured high specificity, minimized nonspecific amplifications, and optimized annealing conditions. Additionally, an internal control primer targeted the human growth hormone (*HGH*) gene. The reference sequence for the *HGH* gene (accession no. NG_042788) was obtained from RefSeq database. Both forward and reverse primer of internal control were also analyzed for specificity and cross-reactivity using the nucleotide BLAST in NCBI database.

### 2.5 Analysis of the HLA-B*57:01 and HLA-B*58:01 alleles using in-house 2-steps PCR-SSP

For the detection of HLA-B*57:01, a 12.5 µL reaction mix was prepared containing 6.25 µL of 2x Quick Tag HS Dye mix (Toyabo, Japan), 2.0 µL of 10 µM specific forward and reverse primers, 1.0 µL of 10 µM internal control forward and reverse primers, and 1.25 µL of sterilized distilled water. Input DNA of 100 ng yielded optimal amplification.

For detection of HLA-B*58:01, a 12.5 µL reaction mix was prepared containing 6.25 µL of 2x Quick Tag HS Dye mix (Toyabo, Japan), 1.0 µL of 10 µM specific forward and reverse primers, 1.0 µL of 10 µM internal control forward and reverse primers and 3.25 µL of sterilized distilled water. Input DNA of 25 ng yielded optimal amplification.

The PCR amplification for both HLA-B*57:01 and HLA-B*58:01 followed this steps: 1 cycle of an initial denaturation at 94 °C for 2 min, followed by 30 cycles of 94 °C for 30 s and extension at 68 °C for 1 min. The reaction was then held at 4 °C. A summary of PCR protocol was presented in Table 1. Following the PCR amplification, the quality of the PCR product was assessed using 2% agarose gel electrophoresis at 100 V for 40 min. The key characteristic of the DNA polymerase in the 2x Quick Tag HS Dye mix that allows a 2-step PCR protocol is its robust activity and fidelity at relatively high annealing/extension temperatures, enabling the annealing and extension steps to be combined as shown in PCR protocol. Also the enzyme’s optimal activity is at 68 °C which allows the PCR cycling to be simplified into two steps with not very high extension temperature. These reduces total PCR run time and reduces handling steps without loss of sensitivity or specificity (Toyobo Co.Ltd, 2025).

**TABLE 1:** Summary of PCR protocol.

Parameter	2-Step PCR-SSP for HLA-B*57:01	2-Step PCR-SSP for HLA-B*58:01
2x Quick Tag HS Dye mix	6.25 µL	6.25 µL
10 µM specific forward + reverse primers	2.0 µL	1.0 µL
10 µM Internal control forward + reverse primers	1.0 µL	1.0 µL
Sterilized distilled water	1.25 µL	3.25 µL
DNA template concentration	100 ng	25 ng
Total reaction volume	12.5 µL	12.5 µL
PCR condition		
Initial denaturation	1 cycle: 94 °C for 2 min 30 cycles: 94 °C for 30 s; 68 °C for 1 min hold: 4 °C	
Cycling steps		

### 2.6 Analysis of the HLA-B*57:01 and HLA-B*58:01 alleles using nanopore sequencing

In this study, the full-length HLA-B was amplified and barcoded using the primers from Matern et al. (2020). The barcoded amplicons were pooled and library preparation was performed using ligation sequencing V14 (SQK-LSK114, Oxford Nanopore Technology, United Kingdom) before loading onto the flow cell, which was the MinION R10.4.1. (FLO-MIN114, Oxford Nanopore Technology, United Kingdom) The sequencer used was the GridION and the sequencing steps followed the recommended protocols provided by Oxford Nanopore Technologies (ONT). Subsequently, the analysis step was performed using an in-house bioinformatics tool. Nanopore sequencing data were processed using an in-house bioinformatics pipeline adapted from Anukul et al. (2023). Nanopore sequencing data were acquired with MinKNOW (Oxford Nanopore Technologies, ONT), which generated POD5 files. Basecalling, demultiplexing, and simultaneous adapter and barcode trimming were performed using Dorado with SUP model (ONT), yielding per-sample FASTQ reads. The reads were aligned to HLA reference sequences for HLA-B downloaded from the IPD-IMGT/HLA database (version 3.48; https://www.ebi.ac.uk/ipd/imgt/hla/) using Minimap2 v2.28 (Li, 2018). Small-variant calling was performed with Clair3, and all variants were phased using WhatsHap v1.6 (Patterson et al., 2015) to produce phased VCF and BAM files. For each haplotype block, supporting reads were extracted from the phased BAM and polished with Racon v1.5.0 (Vaser et al., 2017) to generate allele-specific consensus sequences. Each consensus allele was aligned against the IPD-IMGT/HLA database using BLASTN v2.13 (Altschul et al., 1990), and the best match with the highest bit-score was selected as the final assignment for each HLA allele. This pipeline achieves high-resolution typing (fields 3–4) with low error rates and has demonstrated robust performance across various HLA class I loci beyond HLA-B*57:01 and HLA-B*58:01.

### 2.7 Statistical analysis

PCR-SSP results were compared to sequencing using agreement measured by Cohen’s kappa with 95% CI. With the observed perfect agreement (kappa = 0.81-1.00), it indicates the strange of agreement as almost perfected at a significance level of 0.05 (Wang et al., 2019).

## 3 Results

### 3.1 HLA-B*57:01 test results using the in-house 2-steps PCR-SSP

**FIGURE 1:**
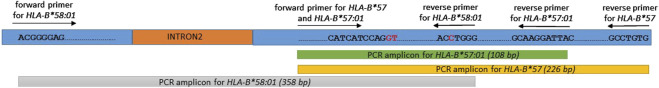
A schematic figure showing primer positions and expected band sizes of HLA-B*57:01 and HLA-B*58:01 alleles detection.

**FIGURE 2:**
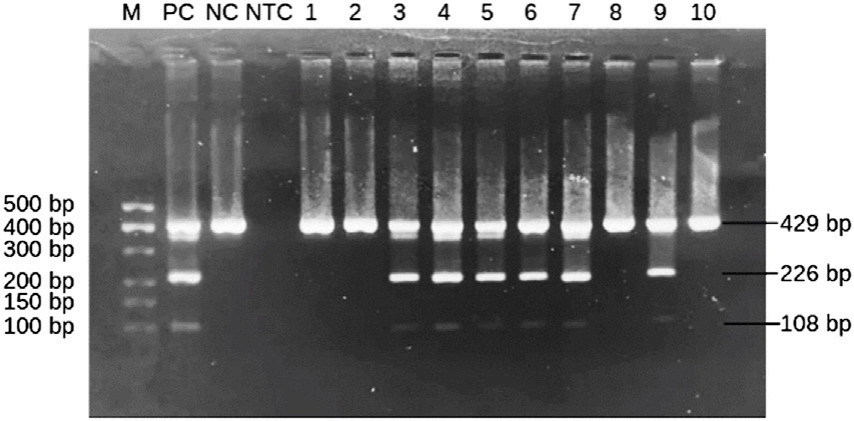
Representative subsets of the total samples analyzed by 2-step PCR-SSP assay for HLA-B*57:01 results on 2% agarose gel. M: marker (100 bp–500 bp); PC: positive control; NC: negative control; NTC: non template control; Lanes 3–7 and 9: HLA-B*57:01-positive samples showing two allele-specific bands at 108 bp and 226 bp plus a 429 bp control band. Lanes 1, 2, 8 and 10: HLA-B*57:01-negative samples with only a control band.

The presence of the HLA-B*57:01 allele is indicated by the appearance of two specific bands for HLA-B*57 at 226 bp and HLA-B*57:01 at 108 bp. Both amplicons were located within exon 3 of *HLA-B* allele based on sequence alignments from the IMGT/HLA database. This dual-band approach reduces the risk of false positives and allows confident identification of the HLA-B*57:01 allele. A schematic figure showing primer positions and expected band sizes of HLA-B*57:01 allele detection is shown in Figure 1. However, in HLA-B*57:01 negative sample, only one band of the internal control at 429 bp will show up (Figure 2). Among the thirty samples tested, nine samples were positive for the HLA-B*57:01, while twenty-one samples were negative (Table 2).

**TABLE 2:** HLA testing results comparison between in-house 2-steps PCR-SSP and nanopore sequencing.

Sample	2-Steps PCR-SSP		Nanopore sequencing		Concordance
	HLA-B*57:01	HLA-B*58:01			
1	Negative	Positive	HLA-B*48:01:01	HLA-B*58:01:01	Yes
2	Negative	Positive	HLA-B*58:01:01	HLA-B*40:06:01	Yes
3	Positive	Negative	HLA-B*57:01:01	HLA-B*35:01:01	Yes
4	Positive	Negative	HLA-B*57:01:01	HLA-B*46:01:01	Yes
5	Positive	Negative	HLA-B*57:01:01	HLA-B*52:01:01	Yes
6	Positive	Negative	HLA-B*57:01:01	HLA-B*27:04:01	Yes
7	Positive	Negative	HLA-B*57:01:01	HLA-B*40:01:02	Yes
8	Negative	Positive	HLA-B*46:01:01	HLA-B*58:01:01	Yes
9	Positive	Negative	HLA-B*57:01:01	HLA-B*40:06:02	Yes
10	Positive	Negative	HLA-B*57:01:01	HLA-B*38:02:01	Yes
11	Negative	Positive	HLA-B*51:01:01	HLA-B*58:01:01	Yes
12	Negative	Positive	HLA-B*58:01:01	HLA-B*46:01:01	Yes
13	Positive	Negative	HLA-B*57:01:01	HLA-B*55:02:01	Yes
14	Negative	Negative	HLA-B*15:02:01	HLA-B*46:01:01	Yes
15	Positive	Negative	HLA-B*13:01:01	HLA-B*57:01:01	Yes
16	Negative	Positive	HLA-B*35:05:01	HLA-B*58:01:01	Yes
17	Negative	Negative	HLA-B*13:01:01	HLA-B*40:01:02	Yes
18	Negative	Positive	HLA-B*40:01:02	HLA-B*58:01:01	Yes
19	Negative	Negative	HLA-B*07:02:01	HLA-B*15:64:01	Yes
20	Negative	Positive	HLA-B*58:01:01	HLA-B*51:01:01	Yes
21	Negative	Negative	HLA-B*40:01:02	HLA-B*38:02:01	Yes
22	Negative	Negative	HLA-B*37:01:01	HLA-B*40:01:02	Yes
23	Negative	Negative	HLA-B*46:01:01	HLA-B*27:04:01	Yes
24	Negative	Negative	HLA-B*15:25:01	HLA-B*51:02:01	Yes
25	Negative	Negative	HLA-B*51:02:01	HLA-B*46:01:01	Yes
26	Negative	Negative	HLA-B*46:01:01	HLA-B*44:03:02	Yes
27	Negative	Negative	HLA-B*46:01:01	HLA-B*35:05:01	Yes
28	Negative	Negative	HLA-B*56:01:01	HLA-B*38:02:01	Yes
29	Negative	Negative	HLA-B*46:01:01		Yes
30	Negative	Negative	HLA-B*40:01:02		Yes

### 3.2 HLA-B*58:01 test results using the in-house 2-steps PCR-SSP

According to the test results and interpretation of the HLA-B*58:01 test using in-house 2-steps PCR-SSP method, it was found that using a DNA concentration of at least 100 ng/μL which similar to the HLA-B*57:01 test, was not suitable for detecting HLA-B*58:01. Therefore, the appropriate concentration for this test was determined to be 25 ng/μL. Notably, the optimal DNA input for HLA-B*58:01 detection was lower (25 ng/μL) compared to HLA-B*57:01 (100 ng/μL). Higher DNA concentrations may result in reduced amplification efficiency for HLA-B*58:01, likely due to PCR inhibition or competition effects between genomic DNA carried non-target alleles, specific primers and internal control primers (Chou et al., 1992; Schrader et al., 2012). Reducing the template concentration can improve primer specificity and amplification efficiency for HLA-B*58:01, yielding clearer and more specific PCR bands.

**FIGURE 3:**
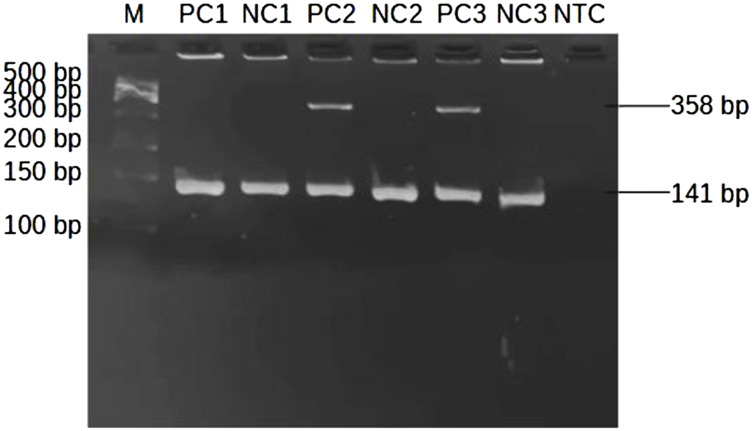
Representative subsets of the total samples analyzed by 2-step PCR-SSP assay for HLA-B*58:01 results on 2% agarose gel. M: marker (100 bp–500 bp); PC1: positive control at 10 ng/μL of DNA; NC1: negative control at 10 ng/μL of DNA; PC2, PC3: positive control at 25 ng/μL of DNA showing a single allele-specific band at 358 bp and a 141 bp control band; NC2, NC3: negative control at 25 ng/μL of DNA showing only a control band; NTC: non template control.

The presence of the HLA-B*58:01 allele is indicated by the appearance of a specific band at 358 bp spans within exon 2-3 of *HLA-B* allele (Figure 1). Sample lacking the HLA-B*58:01 showed only one band of the internal control at 141 bp (Figure 3). Among the thirty samples tested, eight samples were positive for HLA-B*58:01 and twenty-two samples were negative (Table 2).

### 3.3 HLA-B*57:01 and HLA-B*58:01 test results using nanopore sequencing

A total of nine samples were positive for HLA-B*57:01 and eight samples were positive for HLA-B*58:01, while the remaining samples were negative for the HLA-B*57:01 and HLA-B*58:01 alleles (Table 2).

### 3.4 Comparison of HLA-B*57:01 and HLA-B*58:01 results using in-house 2-steps PCR-SSP and nanopore sequencing

The reliability of the in-house 2-step PCR-SSP method was assessed by comparing its results with those from nanopore sequencing (Table 2). The two methods provide different levels of detection resolution. The 2-step PCR-SSP method offers a maximum detection resolution of 2 fields, while the nanopore sequencing method provides a minimum detection resolution of 3 fields. This indicates that the test results obtained from the nanopore sequencing are more detailed and precise, enabling better data differentiation compared to the 2-step PCR-SSP method, which has lower resolution. However, when the results from both methods were compared and statistically examined for consistency using Cohen’s kappa statistic (kappa score), both methods produced a kappa value of 1.00 indicating highly consistent at 2-field resolution. In terms of time consuming and cost per sample of the assay, in-house 2-steps PCR-SSP assay required 1 h PCR hand-on time excluding DNA extraction and agarose gel analysis and reagents costing USD 7 per sample, *versus* 3–5 h and USD 190/sample for Oxford Nanopore sequencing (Anukul et al., 2023).

## 4 Discussion

Typically, the detection of *HLA* genes using the 3-step PCR-SSP method requires at least 2–3 h for the PCR steps (Martin et al., 2005; Olerup and Zetterquist, 1992; Virakul et al., 2010). Our in-house 2-steps PCR-SSP can reduce the PCR step duration to just 1 h. When compared to standard sequencing methods such as Sanger sequencing, Illumina sequencing, PacBio sequencing, and nanopore sequencing, the 2-step PCR-SSP method provides faster result and is more cost-effective in allele-specific testing for pharmacogenomics aspect. In this study, nanopore sequencing was selected because it directly determines haplotype phasing of HLA alleles after sequencing which is the main advantage of nanopore sequencing over other sequencing methods. Several previous reports including our study show high sensitivity, specificity and concordance between PCR-SSP and other HLA typing technologies. De Spiegelaere et al. (2015) presented high concordance between sequence specific PCR with capillary electrophoresis (SSP-PCR CE) and commercial HLA-B SSO Typing Kit, Flow cytometry and qPCR for HLA-B*57:01 typing (De Spiegelaere et al., 2015). Similarly, Nguyen et al. (2017) evaluated the performance of in-house develop SYBR^®^ Green real time PCR for HLA-B*58:01 typing compared to Luminex SSO/SBT/SSP and found it to be highly accurate and low cost. However, our study extends these concordance findings of developed in-house method by comparing PCR-SSP to the more recent nanopore sequencing technology which previously published with high-resolution with very little ambiguity result (Anukul et al., 2023; Ton et al., 2018). Notably, it is important that the 100% concordance between our in-house PCR-SSP and nanopore sequencing only refers to the presence or absence of the particular HLA-B*57:01 and HLA-B*58:01 alleles, not the resolution level. Although the PCR-SSP assay offers the detection at the 2-field level, nanopore sequencing offers higher-resolution genotyping at the 3–4 field level, allowing detection of sub-allelic variants. The difference in resolution levels between nanopore sequencing and PCR-SSP relates to the level of detail in allele typing. Nanopore sequencing provides higher resolution with haplotype phasing and distinguishing sub-allelic variants, which may benefit clinical areas requiring fine allele discrimination such as stem cell transplantation. However, for pharmacogenomic decision-making in current clinical practice, the typing results at 2-field resolution level is sufficient to guide drug hypersensitivity risk assessment (Sukasem, 2009; De Spiegelaere et al., 2015; Nguyen et al., 2017). Therefore, our study’s finding of perfect concordance between PCR-SSP and nanopore sequencing confirms the concordance at 2-field resolution adequately supports clinical pharmacogenomic applications for HLA-B*57:01 and HLA-B*58:01 alleles which makes the cost-effective and rapid PCR-SSP assay suitable for routine use in resource-limited settings. However, nanopore sequencing offers a higher resolution that is useful for research area. Sub-allelic variations of HLA alleles may be linked to more complex or unknown drug hypersensitivity reactions. With this detailed haplotype data, future research may find novel association between sub-allelic variations and adverse drug reactions. However, at this point of study, this study is limited by a relatively small sample size of 30 clinical DNA specimens, which restricts the broad applicability of our findings. To obtain more accurate and reliable comparative results, larger-scale studies with statistically powered and representative sample sizes are essential especially expand to multi-ethnicity cohort to a broader population. We recommend that future research include random sampling strategies based on allele frequency data and extended testing cohorts to confirm these promising results and facilitate broader clinical implementation.

## 5 Conclusion

A comparison of HLA-B*57:01 and HLA-B*58:01 alleles detection results using in-house 2-steps PCR-SSP and nanopore sequencing demonstrated consistent outcomes at 2-field resolution. Consequently, this developed method can be considered for practical application in pharmacogenomics laboratories and it offers a reduced PCR time of 1 h.

## Data Availability

The datasets presented in this article are not readily available because of patient privacy protection. Requests to access the datasets should be directed to Nampeung Anukul at nampeung.a@cmu.ac.th.
